# Intestinal edema induced by LPS-induced endotoxemia is associated with an inflammasome adaptor ASC

**DOI:** 10.1371/journal.pone.0281746

**Published:** 2023-02-17

**Authors:** Toshihiro Yamamoto, Mie Kurata, Naoe Kaneko, Junya Masumoto

**Affiliations:** Department of Pathology, Proteo-Science Center and Graduate School of Medicine, Ehime University, Toon, Ehime, Japan; Toho University Graduate School of Medicine, JAPAN

## Abstract

The apoptosis-associated speck-like protein containing a caspase recruitment domain (ASC)/caspase-1/interleukin(IL)-1β axis, also known as the inflammasome pathway, is indispensable for IL-1β activation in response to various pathogens or own damages. Previously, we developed an NLRP3-inflammasome using a cell-free system and identified ASC targeting drugs; thus, examination of ASC-related histopathology in various diseases could help to provide indications for these drugs. Here, we generated mice deficient only in ASC-protein (ASC-deficient (AD) mice) using CRISPR/Cas9 technology, studied which tissues were most affected, and obtained histopathological images of lipopolysaccharide (LPS)-induced endotoxemia. C57BL/6 wild-type (WT) and (AD) mice were injected intraperitoneally with a lethal dose (50 μg/g) of LPS. Statistical analysis of the survival of C57BL/6 mice and AD mice was performed using the Kaplan–Meier method and the log-rank test. The histopathological findings of multiple tissues from these mice were compared. Acute inflammation (e.g., catarrhal inflammation), along with congestion was observed in the colon of WT mice but not in that of AD mice. Adhesion of neutrophils to capillaries, along with interstitial infiltration, were observed in multiple tissues from WT mice. In AD mice, neutrophil infiltration was less severe but remained evident in the stomach, small intestine, heart, liver, kidney, spleen, and brain. Notably, there was no difference between WT and AD mice with respect to alveolar neutrophil infiltration and interstitial edema. These findings suggest that even though ASC contributes to systemic inflammation, it is dependent on the tissue involved. Intestinal congestion and edema might be good candidates for anti-ASC-targeted therapy.

## Introduction

The apoptosis-associated speck-like protein containing a caspase recruitment domain (ASC) is a component of the inflammasome, which typically consists of NOD-like receptors (NLR)s, ASC, and pro-caspase-1. The inflammasome is indispensable for interleukin (IL)-1β activation in response to various pathogens or own damages [1–3]. Previously, we developed an NLR family pyrin domain containing (NLRP)3-inflammasome in a cell-free system to screen for inflammasome targeting drugs and identified ASC-targeting drugs that suppressed IL-1β secretion from peripheral blood mononuclear cells incubated with lipopolysaccharide (LPS), as well as spontaneous IL-1β secretion from the peripheral blood mononuclear cells of a patient with Muckle-Wells syndrome [4].

If ASC-targeting drugs are to be administered appropriately, it will be important to obtain drug indications for these drugs by analyzing their effects on the ASC-associated histopathology of various diseases. Thus, we generated mice deficient only in ASC-protein (ASC-deficient (AD) mice) using CRISPR/Cas9 technology, studied which tissues were most affected, and examined the histopathology of tissues after LPS-induced endotoxemia, a mouse model of septic shock [5]. Although genomic responses in mouse models poorly mimic human inflammatory diseases [6], a model of endotoxemia could still provide information on the inflammatory pathways involved in sepsis and contribute to preclinical drug development [7].

The pathology of sepsis involves innate immune signaling via pattern recognition receptors (PRRs), which detect various pathogens; such responses play important roles in host tissue damage [8]. Signaling via Toll-like receptors (TLR), especially TLR4, triggers release of inflammatory mediators, which play critical roles in the sepsis model of LPS-induced endotoxemia [9, 10]. NLR is one PRR that senses various intracellular bacterial components and/or damaged self-molecules. The NLRP3 senses various pathogen-associated molecular patterns (PAMPs) and damage-associated molecular pattens (DAMPs), including bacterial RNA, hyaluronan, extracellular ATP, uric acid crystals, amyloid fibrils, and potassium (K^+^) efflux, either directly and/or indirectly [11, 12]. When NLRP3 senses these patterns, it forms an inflammasome with ASC and pro-caspase-1, which is a large molecular-weight platform indispensable for IL-1β processing [13, 14]. Intracellular LPS, which is sensed by caspase-11 independent of TLR4, activates the NLRP3 inflammasome via potassium (K^+^) efflux or direct interaction between caspase-11 and NLRP3 [15–19].

Thus, inflammasome pathways, including ASC, play crucial roles alongside TLR pathways in the pathology of sepsis in the LPS-induced endotoxemia [15–19]. ASC is therefore an attractive target for the treatment of endotoxemia. In this context, several reports have described the role of ASC, including that of the NLRP3-ASC inflammasome, in endotoxin shock. However, most of these studies focused exclusively on the molecular basis of the mechanisms involved, without paying sufficient attention to the histopathology of endotoxemia [20, 21].

Non-coding RNAs (ncRNAs), such as microRNAs (miRNAs), long non-coding RNAs (lncRNAs), and circular RNAs (circRNAs) modulate the various targets of related genes and their functions [22]. Indeed, the *pycard* gene 3’UTR SNP, which is located 3 bp downstream from the stop codon, is an inflammatory response modulator 3 (irm3). The predicted mRNA secondary structure has a long stem without a loop after the SNP containing adenine or a short stem followed by a loop after the SNP containing thymine. The region predicted as an miRNA target or the region itself may regulate inflammatory responses [23]. Although several individual gene-targeted-*pycard*-knockout (KO) strains have been produced, the *pycard* gene containing irm3 was completely deleted from those strains [21, 24, 25].

Thus, in the present study, we generated AD mice using CRISPR/Cas9 technology. Since the AD mice retain all *pycard* genes, they should facilitate histopathological analysis of the role of only ASC protein in LPS-induced endotoxemia. Using the newly developed AD mice, we performed histopathological analysis of tissues after induction of LPS-induced endotoxemia to obtain histopathological images that could be used to examine the effects of anti-ASC targeted therapies.

## Results

### Generation of AD mice

**Fig 1:**
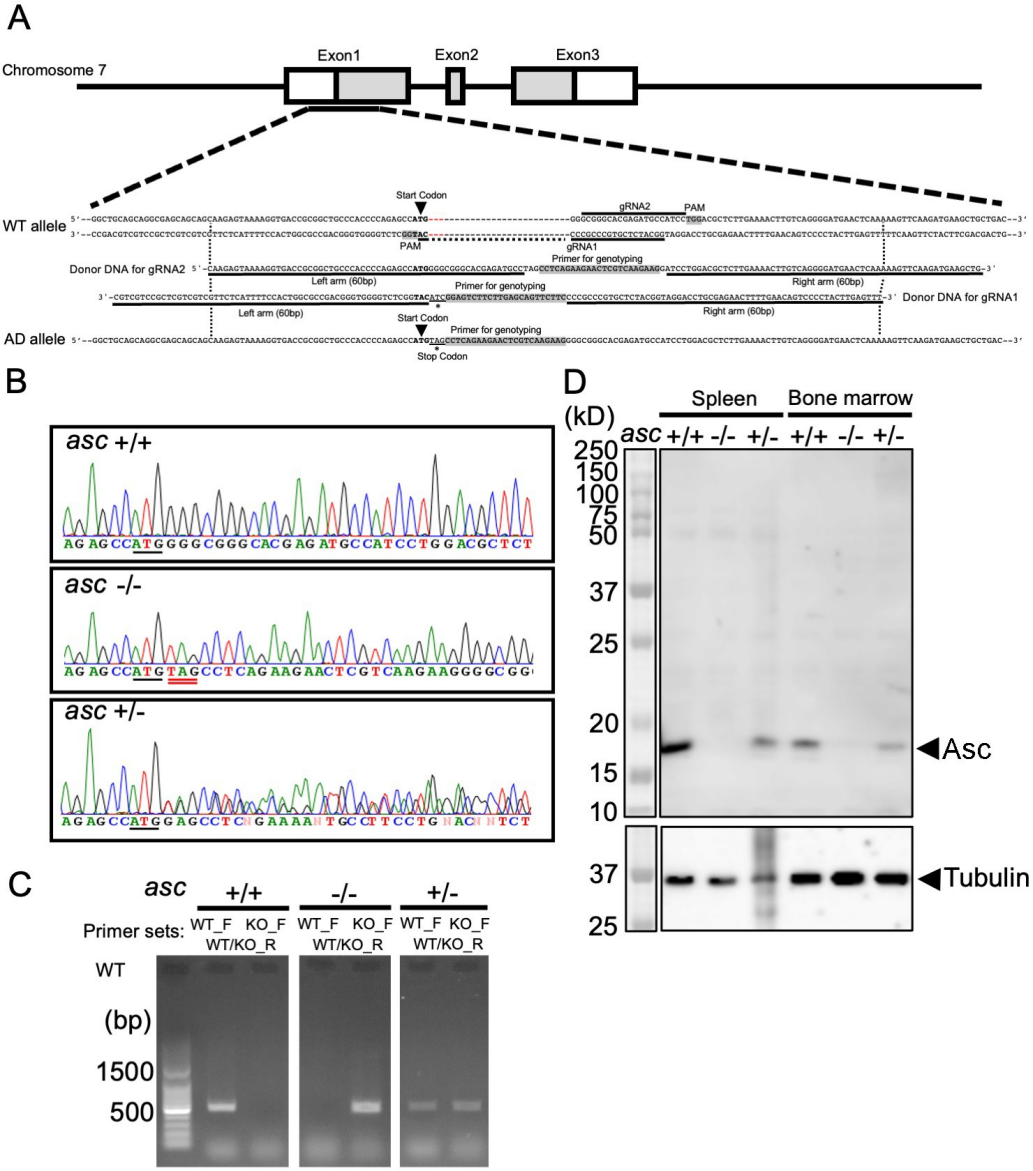
Generation of AD mice. **(A)** Schematic representation of the locus of the *asc/pycard* gene, the doner DNAs for gRNA1 and gRNA2, and the mutated allele. The location of the primer used for PCR is also indicated. **(B)** The edited sequence was confirmed by DNA sequencing using a conventional Sanger method. **(C)** Mutation-specific PCR using the specific primer sets indicated in Table 1. **(D)** Western blot analysis of ASC expression in bone marrow and spleen lysates from wild-type homozygote mice (*asc* +/+), and AD homozygote mice (*asc* -/-) and heterozygote mice (*asc* +/-). Tubulin was used as a loading control.

First, we generated AD mice by inserting the “ochre” stop codon (TAG) after the start codon (ATG) in the coding region of the *pycard* gene (Fig 1A). The Kozak consensus sequence, followed by start codon (agccATGG), was confirmed by Sanger sequencing of DNA from wild-type (WT)(*asc* +/+) mice (Fig 1B; upper panel). We also confirmed successful insertion of a stop codon (TAG) after the start codon (ATG) in AD homozygote mice (Fig 1B; middle panel). Both sequences overlapped in heterozygote mice (Fig 1B; lower panel). Each genotype was distinguishable by PCR using specific primer sets (Fig 1C and Table 1). Western blot analysis revealed that the endogenous ASC protein was expressed in the spleen and bone marrow of WT mice (*asc* +/+), but not in those of AD mice (*asc* -/-) (Fig 1D). The ASC protein was also expressed in heterozygote mice (*asc* +/-), albeit to a lesser extent than in WT mice (Fig 1D). AD mice were born at the expected Mendelian ratio. There were no abnormalities, and the mice appeared healthy when housed under SPF conditions, similar to the *pycard* gene-deficient mice previously described [21, 24, 25].

**Table 1:** Primers used for genotyping.

Name	Sequence
ASC_WT_F	5’-CGCCTGCCCACCCCAGAGCCATGGG-3’
ASC_KO_F	5’-CCTCAGAAGAACTCGTCAAGAAGGGGC-3’
ASC_WT/KO_R	5’-TGGCTGCAGCTGCCACAGCTCCAGACT-3’

### The bone marrow cells of AD mice are unable to secrete IL-1β but they do secrete TNF-α

**Fig 2:**
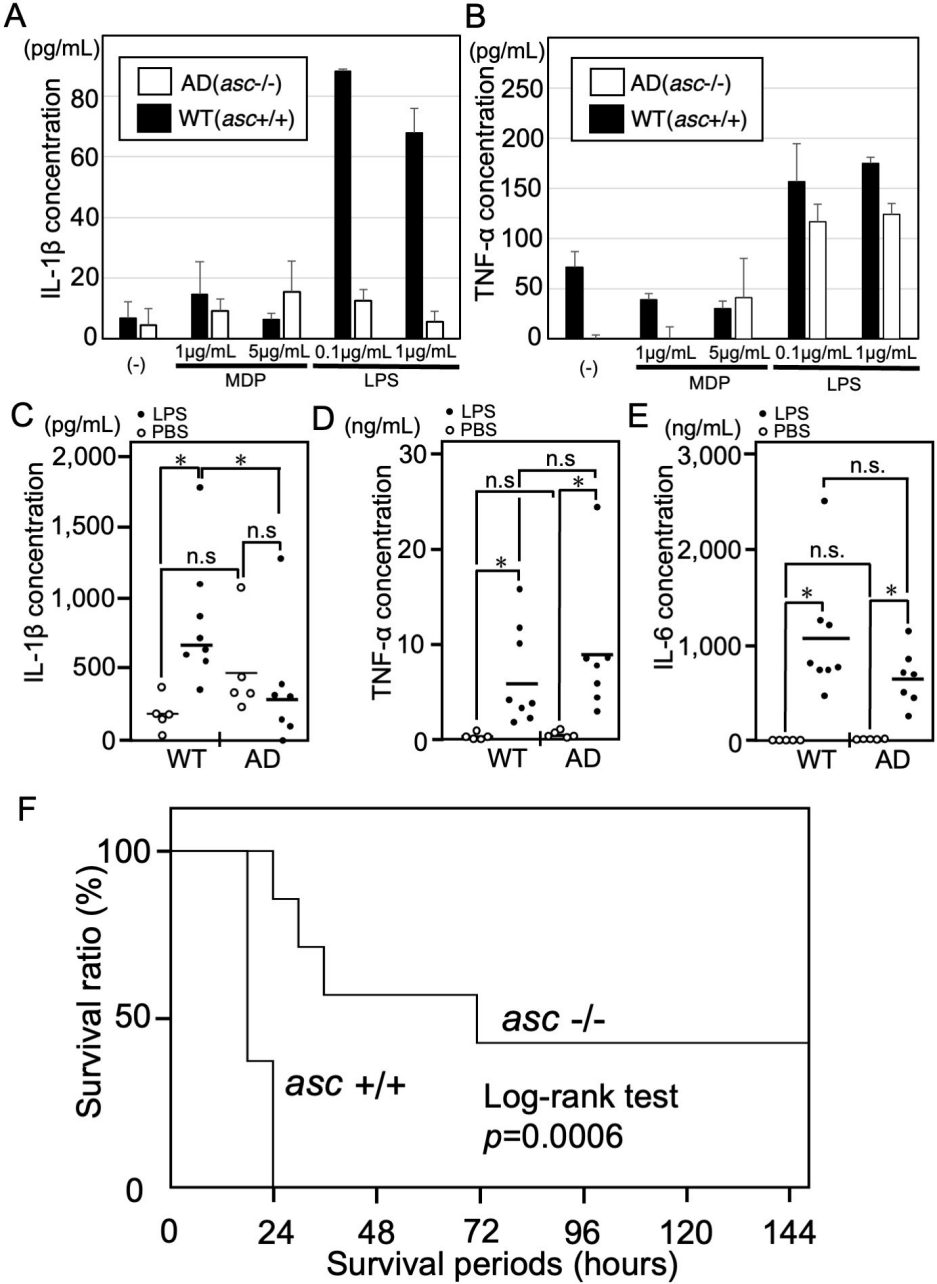
IL-1β secretion is suppressed in AD mice, which survive for longer than WT mice after LPS challenge. **(A, B)** Bone marrow cells (2×10^5^) were incubated for 8 h with the indicated concentrations of MDP or LPS. IL-1β (A) and TNF-α (B) concentrations in culture supernatants were measured by ELISA. Results are presented as the mean SD of triplicate cultures. Data are representative of three independent experiments. **(C–E)** WT and AD mice were injected intraperitoneally with 50 μg/g LPS in PBS (black circles) or PBS alone (white circles). At 4 h after LPS challenge, serum IL-1β (C), TNF-α (D), and IL-6 (E) concentrations were measured by ELISA. Results are plotted and the mean value are presented. *p-value <0.05 was considered significant. **(F)** A Kaplan–Meier survival curve for WT (*asc* +/+) mice (n = 8) and AD (*asc* -/-) mice (n = 7) injected with 50 μg/g LPS. Data were analyzed using the log-rank test (*p* = 0.0006).

To confirm the deficiency of the ASC protein, which is essential for IL-1β processing and activation, in AD mice, we compared IL-1β and TNF-α secretion from AD and WT bone marrow cells in the presence of MDP or LPS (Fig 2A and 2B). Bone marrow cells from AD mice were unable to secrete IL-1β (<20 pg/mL) when incubated with either 0.1 or 1.0 μg/mL LPS for 8 h. By contrast, bone marrow cells from WT mice secreted significant amounts of IL-1β under the same conditions (Fig 2A). However, bone marrow cells from both WT and AD mice secreted TNF-α when incubated with either 0.1 or 1.0 μg/mL LPS for 8 h (Fig 2B). Neither AD nor WT cells secreted IL-1β (<20 pg/mL) and TNF-α (<50 pg/mL) when incubated with 1.0 or 5.0 μg/mL MDP for 8 h (Fig 2A and 2B).

### AD mice express low levels of serum IL-1β, but not TNF-α or IL-6, upon LPS challenge

Both WT and AD mice were challenged intraperitoneally with LPS (50 μg/g body weight) or PBS, and serum concentrations of IL-1β, TNF-α, and IL-6 were measured 4 h later. There was no difference between AD mice with PBS and WT mice with PBS in respect to serum IL-1β concentrations. Serum IL-1β concentrations in AD mice challenged with LPS were significantly lower than those in WT mice challenged with LPS (Fig 2C). However, there was no difference between AD mice and WT mice with respect to serum TNF-α and IL-6 concentrations irrespective of LPS challenge (Fig 2D and 2E).

### AD mice survival for longer than WT mice after LPS-induced sepsis

Upon challenge with LPS, eight out of the eight WT mice and one out of the seven AD mice died within 24 h, while three of the seven AD mice survived for more than 72 h; however, four out of the seven AD mice eventually died (Fig 2F). Kaplan–Meier analysis revealed that AD mice survived for significantly longer than WT mice after LPS-induced sepsis (*p* = 0.0006; Log-rank test) (Fig 2F).

### Histological analysis of tissues from AD and WT mice with LPS-induced sepsis

**Fig 3:**
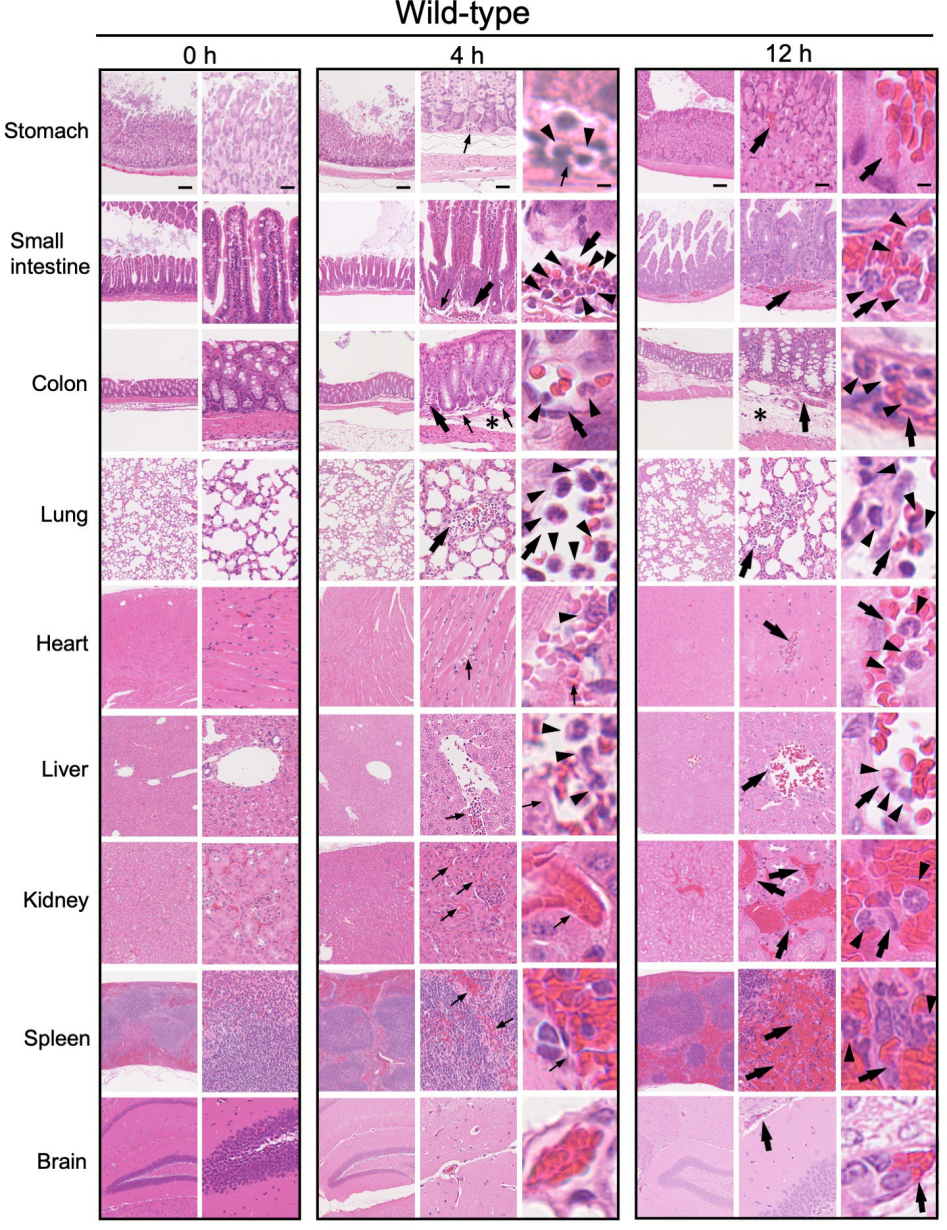
Histopathological images of tissues from WT mice after LPS challenge. Each image is representative of five mice. HE-stained images of the stomach, small intestine, colon, lung, heart, liver, kidney, spleen, and brain tissue from WT mice at 0 (0 h), 4 (4 h), and 12 hours (12 h) after LPS challenge. The asterisk indicates the submucosal edema. Capillary dilation is indicated by small arrows. Congestion is indicated by large arrows. Neutrophils are indicated by arrowheads. Bars, 100 μm (low power view), 25 μm (medium power view) and 5 μm (high power view).

**Fig 4:**
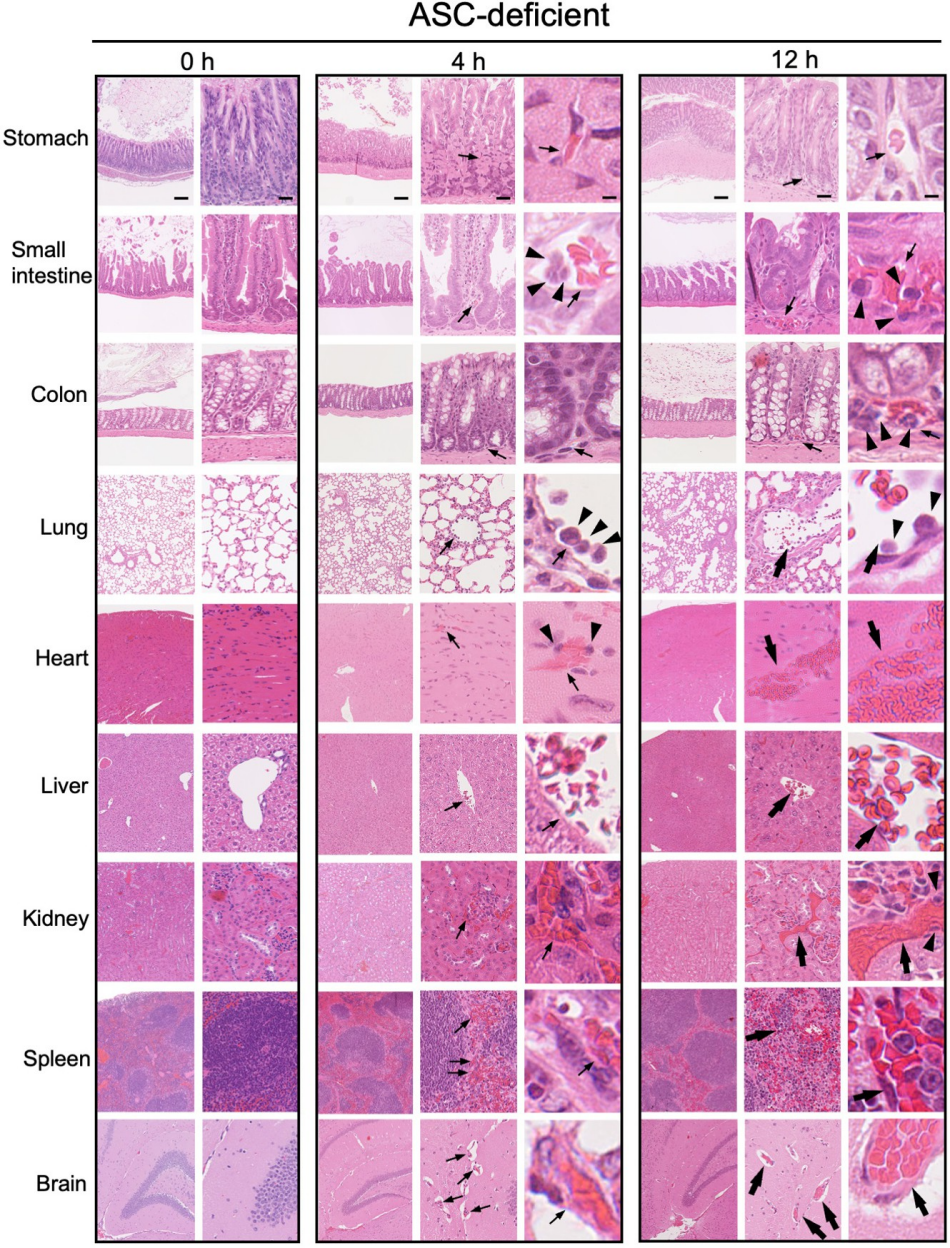
Histopathological images of tissues from AD mice after LPS challenge. Each image is representative of five mice. HE-stained images of the stomach, small intestine, colon, lung, heart, liver, kidney, spleen, and brain tissue from AD mice at 0 (0 h), 4 (4 h), and 12 hours (12 h) after LPS challenge. Capillary dilation is indicated by small arrows. Congestion is indicated by large arrows. Neutrophils are indicated by arrowheads. Bars, 100 μm (low power view), 25 μm (medium power view) and 5 μm (high power view).

To identify the tissues most susceptible to damage by LPS-induced sepsis, we performed histological analysis of tissues from WT and AD mice. We examined hematoxylin and eosin (HE) stained tissues (stomach, small intestine, colon, lung, heart, liver, kidney, spleen, and brain) before and at 4 and 12 h after LPS injection (Figs 3 and 4). At 4 h after LPS injection, congestion (Figs 3 and 4; large arrows) and neutrophil (Figs 3 and 4; arrowheads) adherence to capillary endothelium started to appear in the lungs of both WT and AD mice (Figs 3 and 4). Capillary dilation (Figs 3 and 4; small arrows) appeared in the heart, liver, kidney, and spleen of both WT and AD mice at 4 h (Figs 3 and 4). More congestion (Figs 3 and 4; large arrows) and neutrophil (Figs 3 and 4; arrowheads) recruitment, which is a characteristic of acute inflammation, were observed in the interstitial areas of each tissue at 12 h after LPS injection (Figs 3 and 4). Notably, we observed strong submucosal edema (Fig 3; an asterisk) in the colon of WT mice after LPS injection (Fig 3); this appeared at 4 h after LPS injection but was not seen in AD mice (Fig 4). The histological findings in WT and AD mice are summarized in Tables 2 and 3, respectively.

**Table 2:** Summary of the histological findings in wild-type mice after LPS injection.

Organ and tissuec	4 hours post-LPS injection	12 hours post-LPS injection
Gastrointestinal tract		
Stomach	Submucosal edema	Submucosal edema and congestion
Small intestine	Submucosal edema	Submucosal edema and congestion
Colon	Submucosal edema	Submucosal edema and congestion
Lung	Capillary dilation and neutrophil adhesion	Massive interstitial neutrophil infiltration, endothelial and epithelial dissociation, and congestion
Heart	Neutrophil adhesion and congestion	Neutrophil adhesion and congestion
Liver	Sinusoidal dilatation and congestion	Venous dilation and congestion
Kidney	Capillary dilation	Capillary dilation and congestion
Spleen	Capillary dilation and congestion	Neutrophil infiltration and congestion
Brain	Almost normal	Congestion

**Table 3:** Summary of histological findings in ASC-deficient mice after LPS injection.

Organ and tissue	4 hours post-LPS injection	12 hours post-LPS injection
Gastrointestinal tract		
Stomach	Almost normal	Slight capillary dilation
Small intestine	Capillary dilation	Capillary dilation and neutrophil adhesion
Colon	Almost normal	Capillary dilation and neutrophil adhesion
Lung	Capillary dilation and neutrophil adhesion	Massive interstitial neutrophil infiltration, endothelial and epithelial dissociation, and congestion
Heart	Neutrophil adhesion	Congestion
Liver	Sinusoidal dilatation and congestion	Venous dilation and congestion
Kidney	Capillary dilation	Capillary dilation and congestion
Spleen	Capillary dilation and congestion	Neutrophil infiltration and congestion
Brain	Almost normal	Congestion

### Colonic submucosal edema is significantly less severe in AD mice than in WT mice

**Fig 5:**
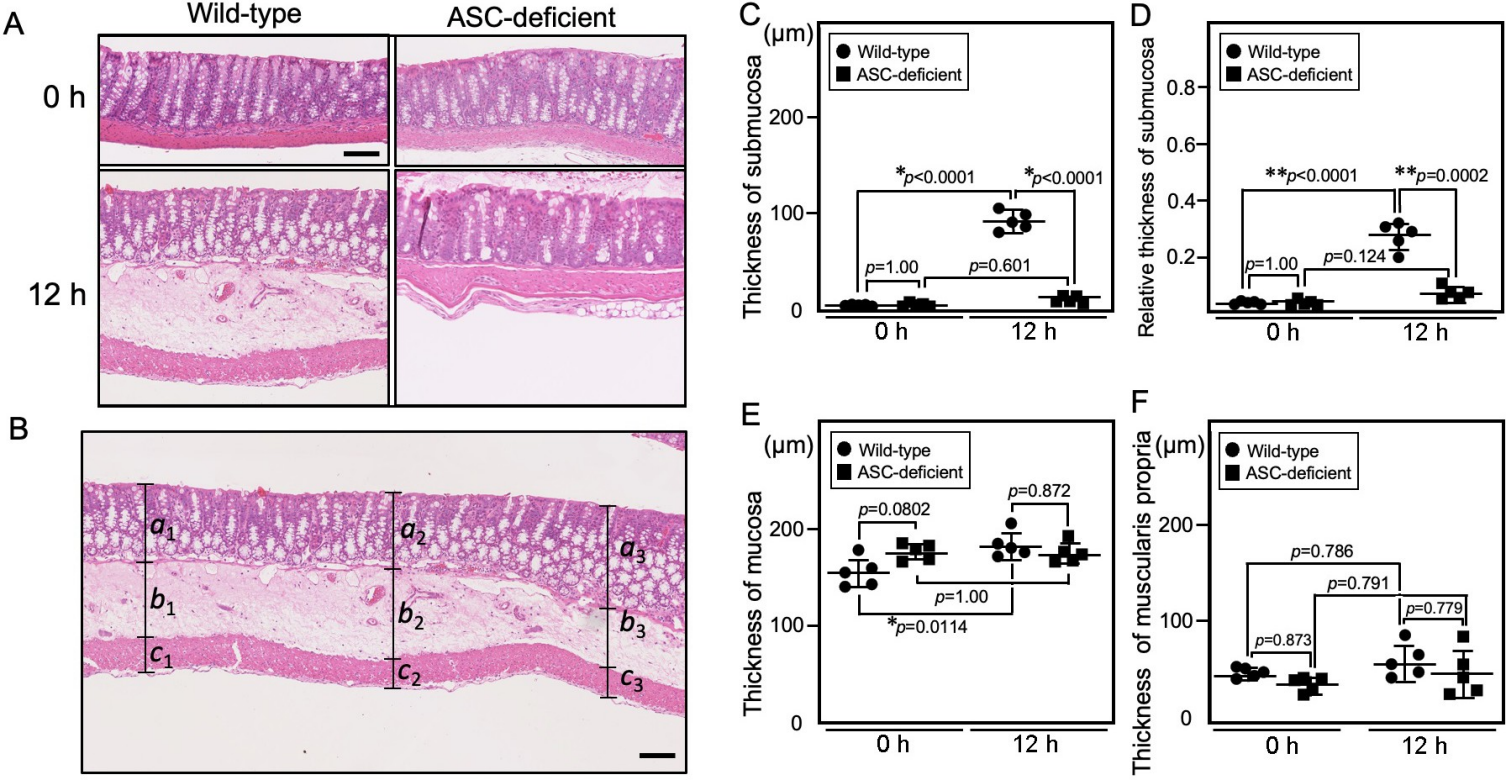
Histopathological comparison of the colonic submucosal edema between WT and AD mice after LPS challenge. Five mice per group were sacrificed. Each image is representative of each group. **(A)** HE staining of the colon before (0 h) and at 12 h after LPS challenge. Bar = 100 μm. (**B**) Illustration of the method used to measure the thickness of the mucosa (*a*), submucosa (*b*), and muscularis propria (*c*) of the colon. The mean thickness was the average of *a*_*1*_ *+ a*_*2*_ + *a*_*3*_. The mean thickness of the submucosa was taken as the average of *b*_*1*_ *+ b*_*2*_ *+ b*_*3*_. The mean thickness of the muscularis propria was taken as the average of *c*_*1*_ *+ c*_*2*_, + *c*_*3*_. Bar = 100 μm. (**C**) The mean thickness of submucosa. (**D**) The mean thickness of the submucosa (*b*) relative to the total thickness of the mucosa/submucosa/muscularis propria (*a+b+c*). (**E**) The mean thickness of mucosa. (**F**) The mean thickness of the muscularis propria. *p-value <0.05. **p-value <0.01.

As described above, submucosal edema in the colon was more severe in WT mice than in AD mice at 12 h post-LPS challenge (Fig 5A). To quantitatively compare submucosal edema between WT mice and AD mice, we measured the thickness of the submucosa at three points within a region of severe edema and averaged these values (Fig 5B). The average thickness of the submucosa in WT mice (95.1 ± 10.0 μm) was significantly greater than that in AD mice (11.4 ± 3.7 μm) at 12 h after LPS challenge (Fig 5C). The average relative thickness of the submucosa in WT mice was 0.263 ± 0.048 in WT mice and 0.062 ± 0.022 in AD mice at 12 h post-LPS challenge (Fig 5D). The average thickness of the mucosa was 182 ± 13.2 μm in WT mice and 173 ± 10.5 μm in AD mice at 12 h post-LPS challenge (Fig 5E). The average thickness of the muscularis propria was 63.1 ± 17.3 μm in WT mice and 50.9 ± 24.1 μm in AD mice at 12 h post-LPS challenge (Fig 5F). There were significant differences between LPS-challenged (12 h) and left-untreated (0 h) WT mice in the thickness of mucosa; however, there were no significant differences between WT and AD mice at either 12 h post-LPS challenge or left untreated (0 h) (Fig 5E).

### Acute interstitial inflammation in the lungs of both AD and WT mice

**Fig 6:**
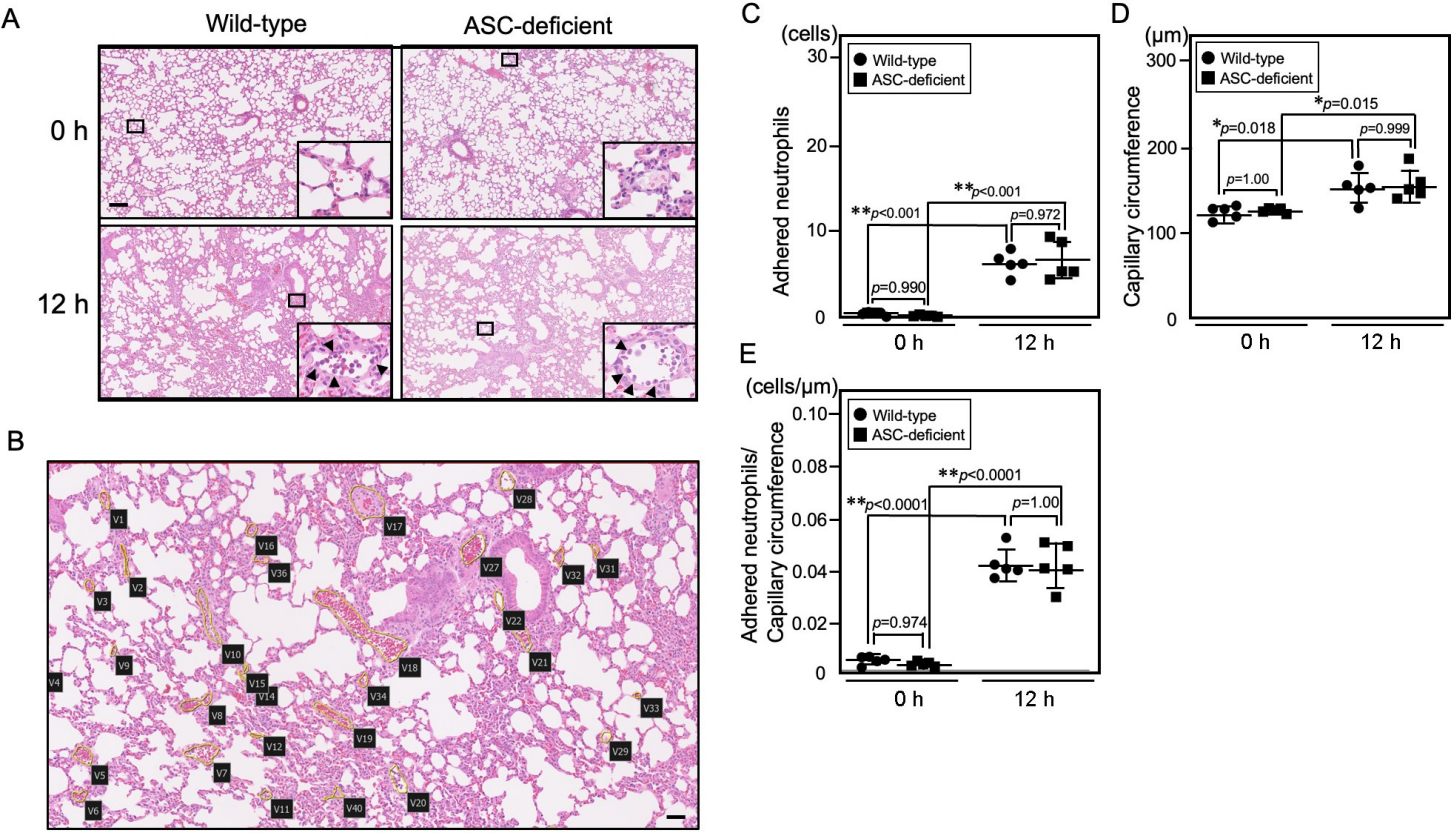
Histopathological comparison of acute lung damage in WT and AD mice after LPS challenge. Five mice per group were sacrificed, and each image is representative of each group. **(A)** HE staining of the lung before (0 h) and at 12 h after LPS challenge. Neutrophils adhering to endothelium are indicated by arrowheads. Bar = 100 μm. **(B)** Illustration of the method used to measure the number of neutrophils adhering to the endothelium of interstitial capillaries, and the mean capillary circumference. The total number of neutrophils in the capillaries marked by a yellow line was counted. In total, 34 capillaries were counted, and the circumference of each was measured. The number of neutrophils per unit of capillary circumference was calculated. Bar = 100 μm. **(C)** The mean number of neutrophils adhering to endothelium of interstitial capillaries. **(D)** The mean capillary circumference. **(E)** Number of neutrophils adhering to the endothelium of interstitial capillaries versus the circumference of the dilated capillaries. Results are expressed as the mean ± standard deviation. *p-value <0.05. **p-value <0.01.

In both WT and AD mice, a high number of neutrophils adhered to the endothelium of dilated interstitial capillaries around the pulmonary alveolus of the lung at 4 h post-LPS injection; these neutrophils were recruited to the interstitial spaces at 12 h post-LPS injection (Figs 3 and 4). To quantitatively compare alveolar inflammation between WT and AD mice (Fig 6A), we counted the number of neutrophils adhered to endothelium in the interstitial area at 12 h post-LPS challenge (Fig 6B). The mean number of neutrophils that adhered to the endothelium in the interstitial area was 6.58 ± 1.37 cells/capillary in WT mice and 6.95 ± 2.33 cells/capillary in AD mice at 12 h post-LPS challenge (Fig 6C). The mean circumference of dilated capillaries in the interstitial area was 156 ± 18.0 μm in WT mice and 159 ± 18.5 μm in AD mice at 12 h post-LPS challenge (Fig 6D). The mean number of neutrophils versus dilated capillary circumference was 0.043 ± 0.006 cells/μm in WT mice and 0.042 ± 0.009 cells/μm in AD mice at 12 h post-LPS challenge (Fig 6E). There were significant differences between LPS-challenged (12 h) and left-untreated (0 h) WT mice and between LPS-challenged (12 h) and left-untreated (0 h) AD mice in the mean number of neutrophils adhering to endothelium of interstitial capillaries, the mean capillary circumference, and number of neutrophils adhering to the endothelium of interstitial capillaries versus the circumference of the dilated capillaries; however, there were no significant differences between WT and AD mice (Fig 6C–6E).

### Expression of IL-1β, TNF-α, and IL-6 in lung and colon from WT and AD mice

**Fig 7:**
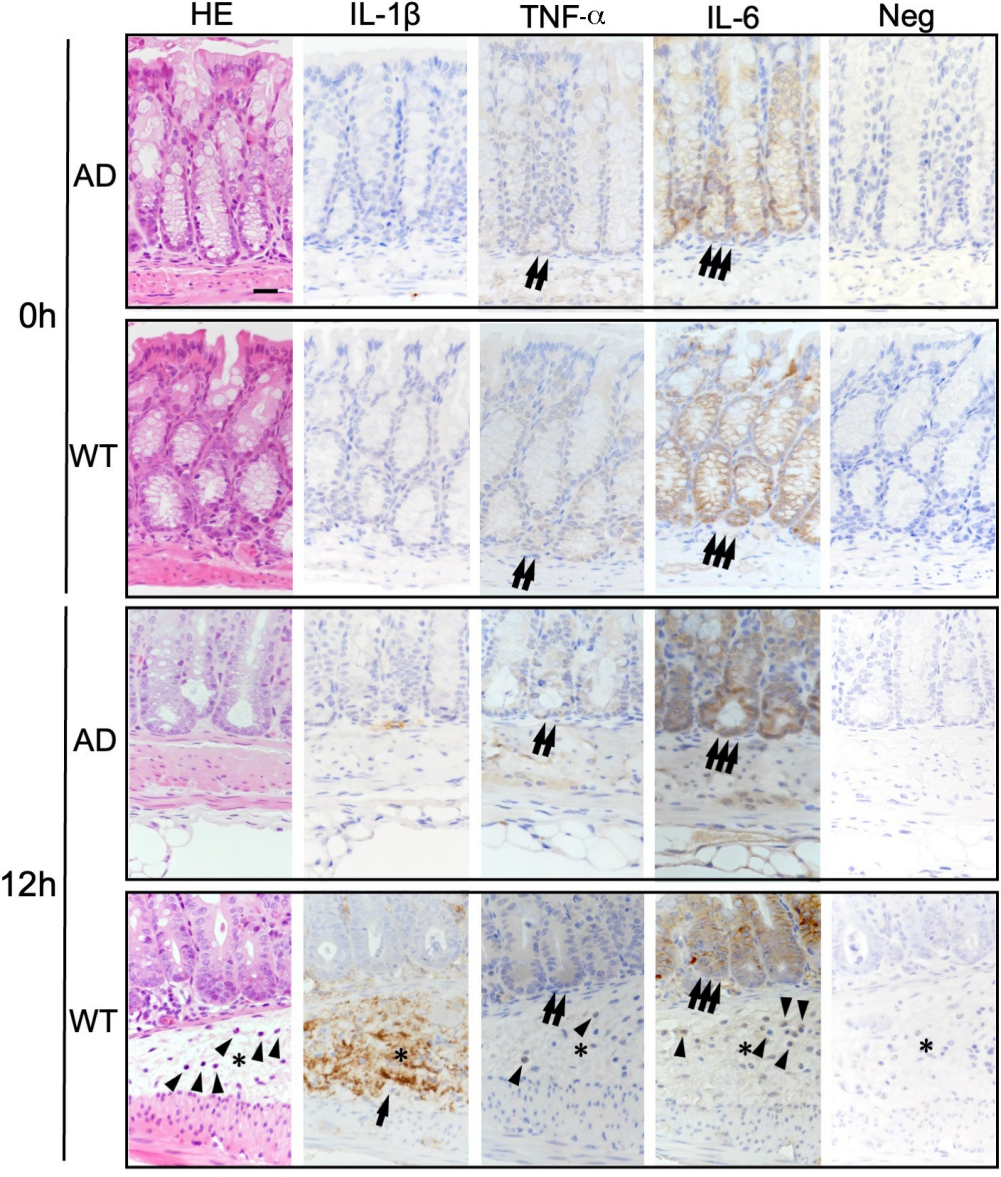
Immunohistochemical comparison of expression of IL-1β, TNF-α, and IL-6 expression in the colon of WT and AD mice after LPS challenge. Formalin-fixed and paraffin-embedded specimens from colon were stained with hematoxylin and eosin (HE). Serial sections were immunostained with antibodies specific for IL-1β, TNF-α, or IL-6. A negative control (Neg) (lacking the primary antibody) is also included. Positive staining for IL-1β, TNF-α, and IL-6 is indicated by an arrow, two arrows, and three arrows, respectively. Asterisks indicate the submucosal edema. Neutrophils are indicated by arrowheads. 0 h, before LPS challenge; 12 h, 12 h after LPS challenge. AD, ASC-deficient; WT, wild-type. Bar = 100 μm.

**Fig 8:**
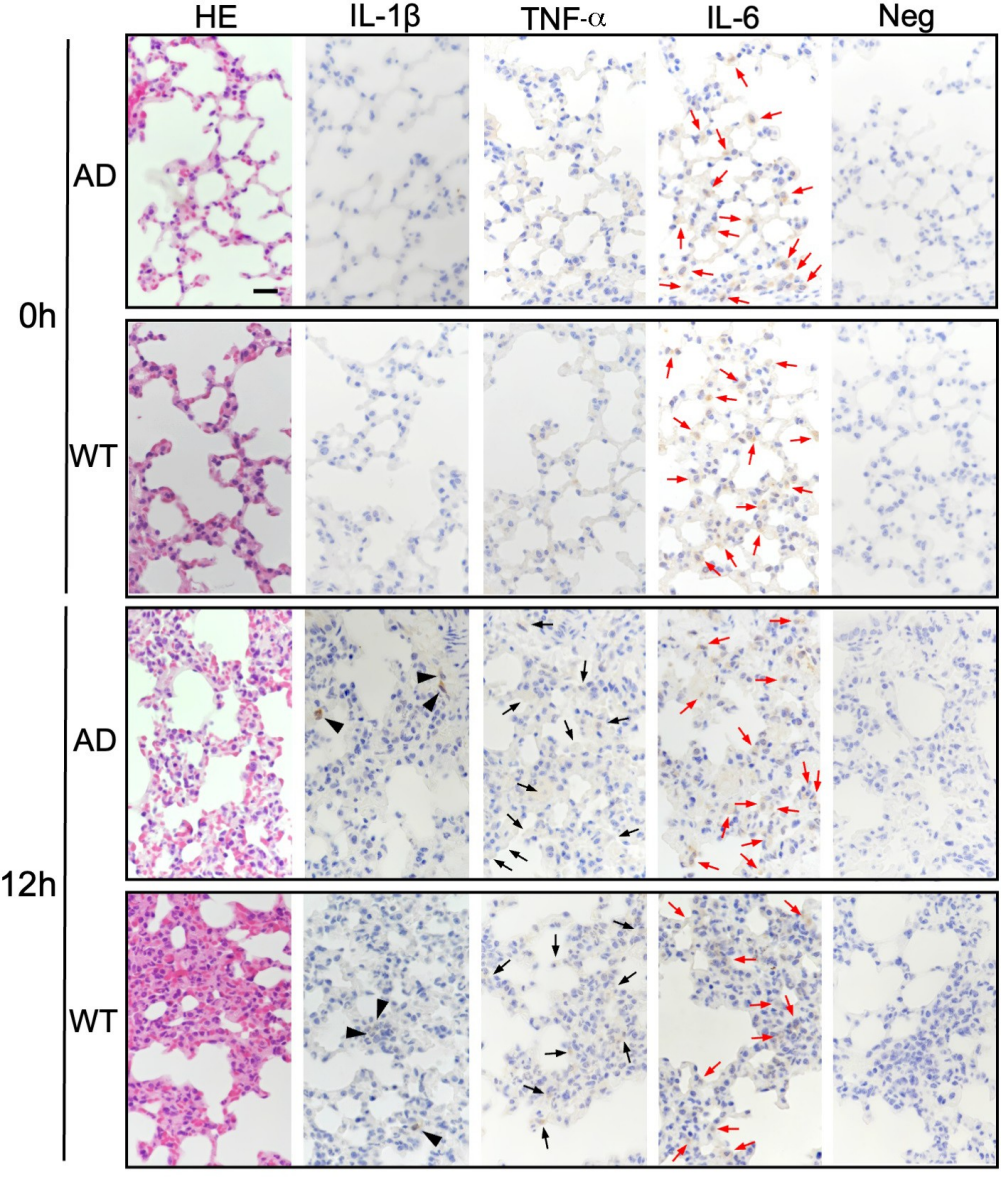
Immunohistochemical comparison of IL-1β, TNF-α, and IL-6 expression in the lungs of WT and AD mice after LPS challenge. Formalin-fixed and paraffin-embedded specimens from lung were stained with hematoxylin and eosin (HE). Serial sections were immunostained with antibodies specific for IL-1β, TNF-α, or IL-6. A negative control (Neg) (lacking the primary antibody) is also included. Positive staining for IL-1β, TNF-α, and IL-6 is indicated by arrowheads, black arrows, and red arrows, respectively. 0 h, before LPS challenge; 12 h, 12 h after LPS challenge. AD, ASC-deficient; WT, wild-type. Bar = 100 μm.

Finally, we performed immunohistochemical analysis to confirm whether expression of IL-1β, TNF-α, and IL-6 was related to colonic submucosal edema and/or acute lung damage (Figs 7 and 8).

In the colon, positive staining for IL-1β was observed widely in interstitial edematous tissue in the colon submucosa (Fig 7; the arrow) of WT mice at 12 h after LPS challenge; no (or weakly positive) staining was observed in the submucosa of AD mice (Fig 7). Positive TNF-α (Fig 7; two arrows) and IL-6 (Fig 7; three arrows) staining were observed in the crypt epithelium of both WT and AD mice before LPS challenge (0 h) (Fig 7). Strong positive TNF-α (Fig 7; two arrows) and IL-6 (Fig 7; three arrows) staining was observed in crypt and glandular epithelium at 12 h after LPS challenge (Fig 7).

In the lung, several infiltrated neutrophils (Fig 8; arrowheads) in the alveolar interstitial tissue of both WT and AD mice were positive for IL-1β at 12 h after LPS challenge, but not before LPS challenge (0 h) (Fig 8). Positive staining for TNF-α (Fig 8; black arrows) was observed in the alveolar epithelium of both WT and AD mice at 12 h after LPS challenge but not before LPS challenge (0 h), and positive staining for IL-6 was observed in the alveolar epithelium at 12 h after LPS challenge; weaker (although still positive) staining was also observed in the alveolar epithelium before LPS challenge (Fig 8).

## Discussion

Inflammasomes are responsible for various inflammatory diseases, which are referred to as inflammasomopathies [26]. Since ASC is an inflammasome component that acts as an adaptor between NLR and pro-caspase-1, ASC is an attractive therapy for the treatment of inflammasomopathies. In this context, we developed several inflammasomes in a cell-free system for drug discovery, and identified small anti-ASC targeting compounds [4, 27]. In this study, we examined ASC-dependent histopathology of tissues with LPS-induced endotoxemia, which could be useful for examining the effects of anti-ASC targeted drugs.

Sepsis is a life-threatening disease caused by bacterial infection [28]. Despite considerable experimental and clinical research during the last three decades, little progress has been made in altering the course and outcome of this syndrome. Although the results of a meta-analysis showed a weak correlation between endotoxemia and Gram-negative bacteremia [29], mouse endotoxemia could be useful as a model of the acute inflammatory response associated with sepsis [7]. Therefore, we used the conventional LPS-induced endotoxemia in mouse model.

First, AD mice were generated by inserting an “ochre” stop codon (TAG) immediately after the start codon (ATG), which was confirmed by sequencing and mutation-specific PCR (Fig 1A–1C). Confirmation that *asc* -/- mice and heterozygote (*asc* +/-) mice showed no or lower, respectively, expression than WT homozygote (*asc* +/+) mice led us to conclude that we had successfully generated AD mice (Fig 1D). Consistent with a previous study on *pycard* gene-KO mice [21, 24, 25], the AD mice generated herein did not secrete IL-1β in response to LPS challenge (Fig 2A–2E). In addition, the AD mice survived for significantly longer than WT mice after LPS challenge, even four out of the seven AD mice eventually died (Fig 2F). Thus, we inferred that the AD mice were almost identical or slight sensitive to *pycard* gene-KO mice in terms of their response to LPS.

Second, to identify ASC-dependent specific histopathological features, histopathological findings in multiple tissues from WT and AD mice were compared (Figs 3 and 4). The results from WT mice revealed acute inflammation (i.e., catarrhal inflammation) in gastrointestinal tissue, in which dilated capillaries were associated with interstitial edema without neutrophil recruitment; however, these features were almost absent from AD mice (Figs 3 and 4). In particular, submucosal edema and congestion in the colon were markedly less severe in AD mice than in WT mice (Figs 5A–5F and 7). A previous study reported that the LPS activates the caspase-11-driven non-canonical inflammasome pathway, resulting in capillary permeability, and that a caspase-11-defect in mice results in reduced endothelial permeability and increased survival upon LPS challenge [30]. Thus, intestinal edema may be highly dependent on ASC. Our finding that strong IL-1β expression (indicated by arrow), which was not seen in AD mice, in the submucosal interstitial edematous tissue (indicated by an asterisk) of WT mice at 12 h after LPS challenge (Fig 7) supports the above hypothesis although TNF-α, IL-6 or the other cytokines are thought to be important.

Microscopic analyses revealed adherence of many neutrophils to the endothelium of dilated interstitial capillaries following acute lung injury (Fig 6A–6E). Neutrophils were recruited into the interstitial spaces not only in WT mice but also in AD mice (Figs 6A and 8). Consistent with our findings, acute lung injury is lethal in mice [31, 32] and a major cause of death in human sepsis patients [33, 34]. Increased neutrophil adhesion and recruitment to microvessels is dependent on TLR4 signaling because neither neutrophil adhesion nor recruitment is observed in TLR4 KO mice in response to LPS [35]. Caspase-11 activated by intracellular endotoxin LPS cleaves Gasdermin D into polypeptides, which form a nanopore in the cytoplasmic membrane [12, 15–17, 36]; this is accompanied by pyroptotic cell death and NLRP3 inflammasome-dependent IL-1β secretion [12, 36–38]. Because IL-1β expression was limited in a few populations of neutrophils (indicated by arrowheads) in the alveolar interstitial tissue of the lung, even at 12 h after LPS challenge (Fig 8), the contribution of the inflammasome might be limited. Expression of TNF-α (indicated by black arrows) and IL-6 (indicated by red arrows) (Fig 8), both of which were downstream cytokines of TLR4, in alveolar endothelial cells was more pronounced at 12 h after LPS challenge than before LPS challenges (0 h) (Fig 8). These data suggest that acute lung injury accompanied by neutrophil infiltration may be related to complex mechanisms involving redundant signaling, and that acute lung injury accompanied by neutrophil infiltration are relatively less associate with ASC.

The study has several limitations. In clinical practice, since sepsis in humans is rarely caused by bacteria that express LPS [39], the findings obtained using the mouse model may not be applicable to human sepsis. Indeed, there are substantial differences between mouse responses and human responses to sepsis at the genomic level [6]. Thus, the LPS-induced sepsis model is only applicable to a subset of human patients with fulminant conditions [40]. Some contamination may be present in biologically purified samples. The LPS used in this study was not chemically-synthesized; rather, it was biologically purified from *Escherichia coli*. Thus, other biologically functional ligands present in the LPS fraction, such as cell wall components, lipids, and DNA/RNA, which are known to modulate host immune responses [41, 42], could have confounded the results.

In summary, we developed mice deficient only in ASC-protein, that were almost identical or slight sensitive to *pycard* gene-KO mice in terms of their response to LPS. Our data suggest that even though ASC contributes to systemic inflammation, the contribution of ASC depends on the tissue involved, and that intestinal congestion and edema might be good candidates for anti-ASC-targeted therapy.

## Materials and methods

### Generation of murine AD mice

The murine AD mouse was generated using the CRISPR/Cas9 method. To generate a mouse lacking expression of the ASC gene, which is encoded by the *pycard* gene on chromosome 7, an “ochre” stop codon (TAG) was inserted into the coding region. The Optimized CRISPR Design web tool (Massachusetts Institute of Technology, Zhang Lab, http://crispr.mit.edu/) was used to design two single guide RNAs (*pycard*-gRNA1: 5’-GGC AUC UCG UGC CCG CCC CA-3’ and *pycard* -gRNA2: 5’-GCG GGC ACG AGA UGC CAU CC-3’) to target the relevant sequence in exon 1 of the mouse *pycard* gene. Plasmids expressing hCas9 and each of the designed sgRNAs (pCAG-Cas9-*pycard*-gRNA1 and pCAG-Cas9-*pycard*-gRNA1) were prepared by ligating the oligonucleotides into the Bbs I site downstream of the U6 promoter in the pCAG-Cas9-gRNA expression vector, in which expression of Cas9 is regulated by the CAG promoter and gRNA expression by the U6 promoter. To validate the efficiency of the sequence-targeted double-strand breaks induced by sgRNA and hCas9, reconstitution of green fluorescence was examined by homology-dependent repair of EGFP using a pCMV-dasherGxxFP plasmid [43]. To construct the pCMV-dasherGxxFP plasmid, the N-terminal and C-terminal dasherGFP sequences (DNA2.0 Inc. Menlo Park CA) were PCR-amplified and placed under control of the CMV promoter; a genomic fragment (approximately 500 bp) containing exon 1 of the *pycard* gene was then inserted between the dasherGFP fragments. Next, pCAG-dasherGxxFP was mixed with pCAG-Cas9-*pycard*-gRNA1 or pCAG-Cas9-*pycard*-gRNA2 and transfected into HEK293T cells. EGFP fluorescence was examined under a fluorescent microscope at 48 h post-transfection. Single-strand oligo-DNAs (ssODNs for *pycard*-gRNA1 and ssODNs for *pycard*-gRNA2; Fig 1A), which include a stop codon, a primer genotyping sequence, and 60 bp homologous left and right arms identical to the upstream and downstream sequences of the fragment to be inserted into the mouse genome sequence, were designed to introduce a stop codon into the coding region in exon 1 of the *pycard* gene; this oligo-DNA was synthesized by Ultramer oligos Integrated DNA Technologies, Inc., and used as donor DNA (Fig 1A).

Fertilized eggs isolated from C57BL/6N mice were injected with 1–2 pl (5 ng/μL) of the pCAG-Cas9-*pycard*-gRNA1 or pCAG-Cas9-*pycard*-gRNA1 plasmid, along with donor DNA ssODNs for *pycard*-gRNA1 or ssODNs for *pycard*-gRNA2. Injected eggs were cultivated and transferred to the oviducts of pseudopregnant mice to generate F0 mutant mice.

To identify the mutant mice, the genomic region containing exon 1 of the *pycard* gene was amplified from the genomic DNA of each F0 mouse using the following primer set: targetF: 5’ -CTC CAC CTA GTT TCT TCA GCC TAG CC- 3’and targetR: 5’ -GTA GAC GAA CAA GGG GAC ACA CTC ACC- 3’. Amplified fragments were direct-sequenced using primer targetF. Twenty-eight mice were obtained from pCAG-Cas9-*pycard*-gRNA1-injected eggs, and the insertion stop codon generated by homology-directed repair was observed in seven mice. Nine mice were obtained from pCAG-Cas9-*pycard*-gRNA2-injected eggs, and insertion of the stop codon by homology-directed repair was observed in one mouse. Six F0 mice (F0-8, F0-10, F0-11, F0-16, F0-18, and F0-19) were mated with C57BL/6N mice, and insertion of the stop codon in exon 1 of the *pycard* gene was analyzed in F1 pups by direct sequencing, as described above. As a result, an allele containing the inserted stop codon was transmitted to the germline of F1 mice derived from F0-16, F0-18, and F0-19 mice, and KO mouse lines were established and supplied by Transgenic Inc. (Fukuoka, Japan). For further study, mice derived from F0-16 and F0-18 were used. Genotyping PCR was performed using WT mice specific forward primer “ASC_WT_F” or AD mice specific forward primer ASC_KO_F and reverse primer ASC_WT/KO_R (Table 1) under following PCR condition; (1) an initial denaturation at 96°C for 5 minutes; (2) 30 seconds of denaturation at 95°C, 1 minutes of annealing at 60°C and 3 minutes of extension at 72°C; (3) and 10 minutes of extension at 72°C using Taq DNA Polymerase (D4545) (Sigma-Aldrich, St. Louis, MO, USA). All mice used in this study were maintained under specific pathogen-free (SPF) conditions. All experiments were performed under protocols approved by the Animal Care Unit Committee of the Ehime University, Ehime, Japan (No.37U4-16) and in accordance with international and Ehime University guidelines for animal experiments.

### LPS-induced endotoxemia

C57BL/6 mice were purchased from CLEA Japan (Tokyo, Japan). A total of 73 mice were used in this study. C57BL/6 WT (*asc* +/+) and AD mice *(asc* -/-*)* mice were housed in standard cages with paper-chips, and maintained under constant ambient temperature (22°C) and humidity (45%) with a 12 h light and dark cycle. Four mice were housed in each cage. All animals had free access to tap water and the assigned diet. All research staffs received special training in animal care and handling provided by Ehime University. Each mouse was injected intraperitoneally with 50 mg/kg of LPS in PBS, or with PBS alone or left untreated. The mice were then monitored (every 4 h for 7 days) by two investigators. Animal health and behavior were monitored twice a day. Humane endpoints were set before the experiment, including reduced dying (depressive looking, low body temperature), and organ failure (decreasing breathing, cyanosis, severe vomiting). Once the preceding symptoms were observed, the mice were euthanized immediately by CO_2_ inhalation. No animal died before meeting the criteria for euthanasia, and the mice were euthanized within 7 days after the experiment (again by CO_2_ inhalation). The experiment was performed from 2020 March to 2020 July. All experiments were performed under protocols approved by the Animal Care Unit Committee of the Ehime University, Ehime, Japan (No.37U4-16) and in accordance with international and Ehime University guidelines for animal experiments.

### Reagents

LPS from *Escherichia coli* (serotype O111:B4, prepared by phenol-chloroform-petroleum ether extraction, catalogue number L2630) and muramyl dipeptide (N-Acetylmuramyl-L-Alanyl-D-Isoglutamine (MDP); catalogue number A9519), a component of bacterial cell wall peptidoglycan, were purchased from Sigma (St. Louis, MO). The monoclonal rat anti-mouse ASC antibody used for the experiments has been described previously [25].

### Assay to measure cytokine production by bone marrow cells

Bone marrow cells were isolated from mice and cultured in 48-well flat-bottom plates (BD Biosciences, San Jose, CA) containing RPMI1640 supplemented with 10% FBS. Cells (final cell density = 1 × 10^6^/ml) were treated (or not) with MDP or LPS for 8 h at 37˚C/5% CO_2_. The concentrations of IL-1β and tumor necrosing factor (TNF)-α in the culture supernatant were measured in an enzyme-linked immunosorbent assay (ELISA) according to the manufacturer’s instructions (BD Biosciences).

### Immunohistochemistry

Immunohistochemical analysis was carried out using the anti-mouse IL-1β rabbit polyclonal antibody (ab205924) (Abcam; Cambridge, UK). An anti-TNF-α rabbit polyclonal antibody (26405-1-AP) and an anti-IL-6 rabbit polyclonal antibody (21865-1-AP) were purchased from Proteintech (Rosemont, IL, USA). Sections (3–5 μm thick) were cut from formalin-fixed paraffin-embedded tissues, deparaffinized in xylene and rehydrated through a decreasing concentration of ethanol solutions. Endogenous peroxidase activity was blocked by addition of 0.3% H_2_O_2_ in methanol for 30 min. Before immunostaining, antigen retrieval was carried out by heating tissue sections in a microwave in 10 mmol/L Tris-HCl buffer (pH 8.0) containing 1 mmol/L ethylenediaminetetraacetic acid (EDTA). Sections were blocked with 1% normal bovine serum in 50 mmol/L Tris-buffered saline (TBS) (pH 7.6) and then incubated with primary antibodies diluted in blocking buffer. Binding was detected using EnVision+ Rabbit/HRP (Dako, Carpinteria, CA, USA), and positive signals were revealed by the addition of diaminobenzidine tetrahydrochloride. Tissue sections were counterstained with hematoxylin and then mounted in Entellan new (Sigma-Aldrich, St. Louis, MO, USA).

### Statistical analysis

Statistical analyses were done using GraphPad Prism Version 9.4.1 (GraphPad Software, San Diego, CA). All results are presented as the mean ± standard deviation (SD) from three independent experiments. Comparisons between multiple groups were made using one-way ANOVA. Survival was analyzed using the Kaplan–Meier method, and data were analyzed using the log-rank test. Differences were considered significant at *p* < 0.05.

## Supporting information

S1 Raw images (PDF)
