# Supplementary material for: Intestinal edema induced by LPS-induced endotoxemia is associated with an inflammasome adaptor ASC
Source: PLoS One. 2023 Feb 17;18(2):e0281746. doi: 10.1371/journal.pone.0281746 (PMC9937502; doi:10.1371/journal.pone.0281746)
Supplement: S1 Raw images — (PDF) [file pone.0281746.s001.pdf]

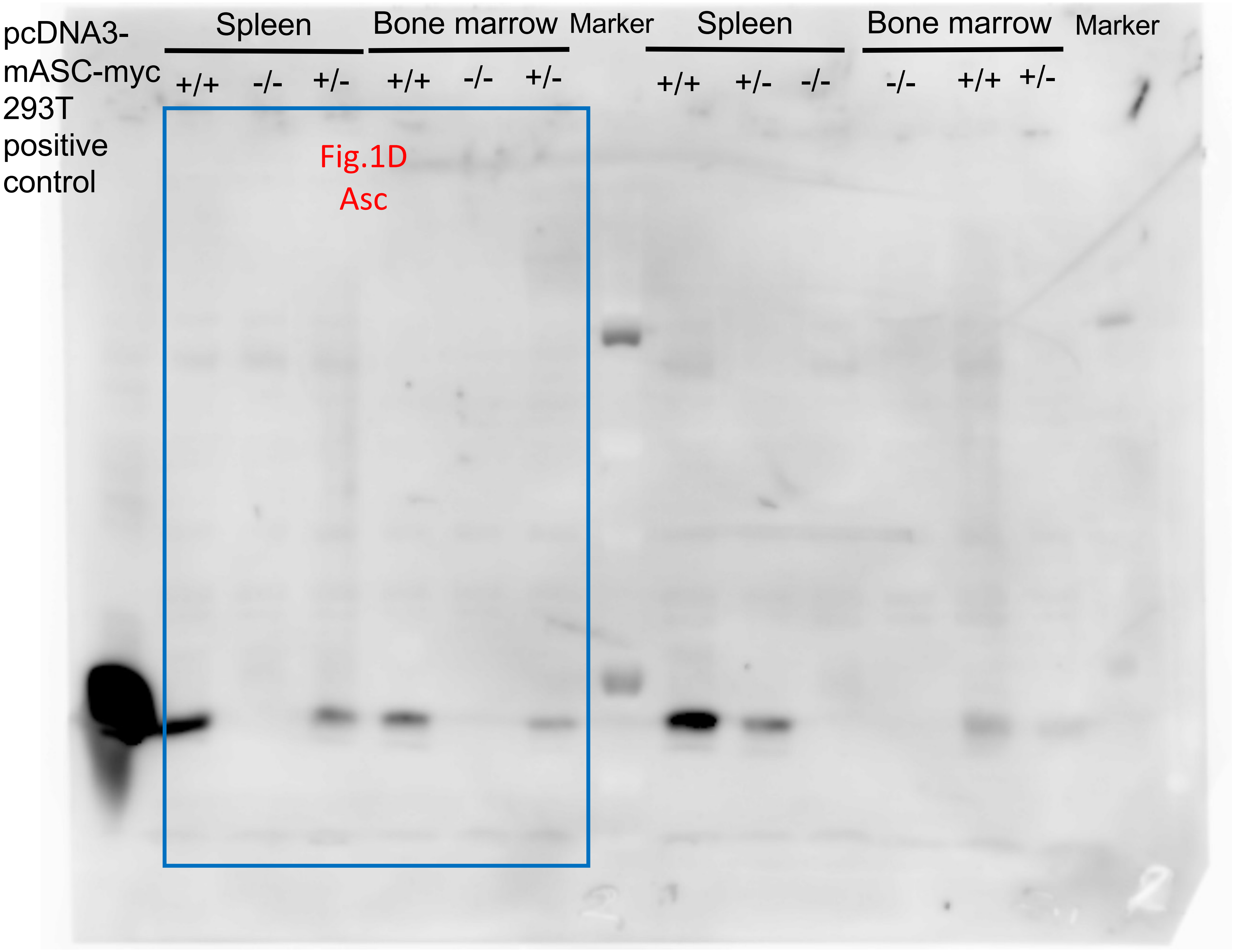
pcDNA3-
mASC-myc
293T
positive
control
Spleen
Bone marrow
Marker
Spleen
Bone marrow
Marker
+/+
-/-
+/-
+/+
-/-
+/-
+/+
+/-
-/-
-/-
+/+
+/-
Fig.1D
Asc

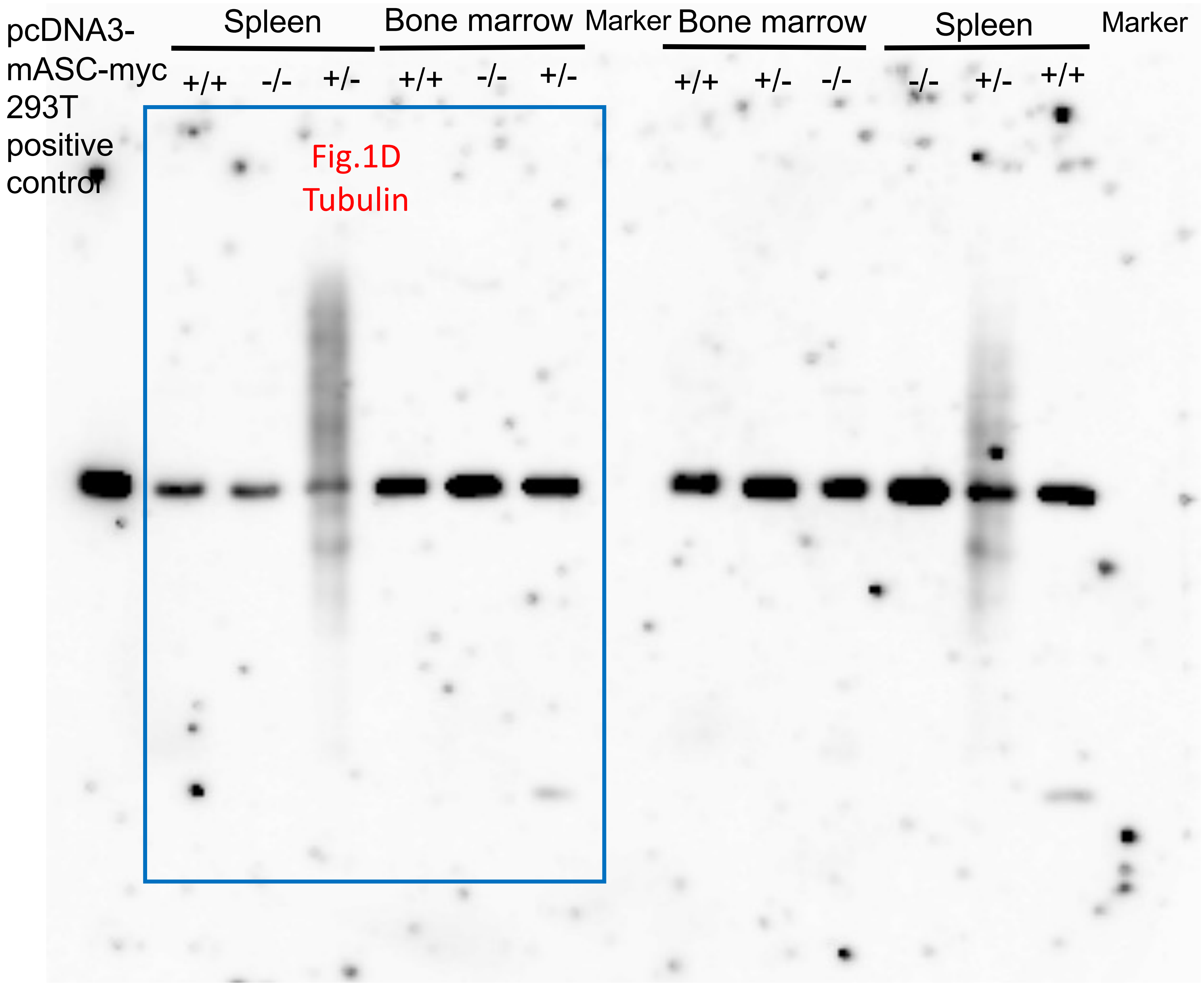
pcDNA3-
mASC-myc
293T
positive
control
Spleen
Bone marrow
Marker
Bone marrow
Spleen
Marker
+/+
-/-
+/-
+/+
-/-
+/-
+/+
+/-
-/-
-/-
+/-
+/+
Fig.1D
Tubulin

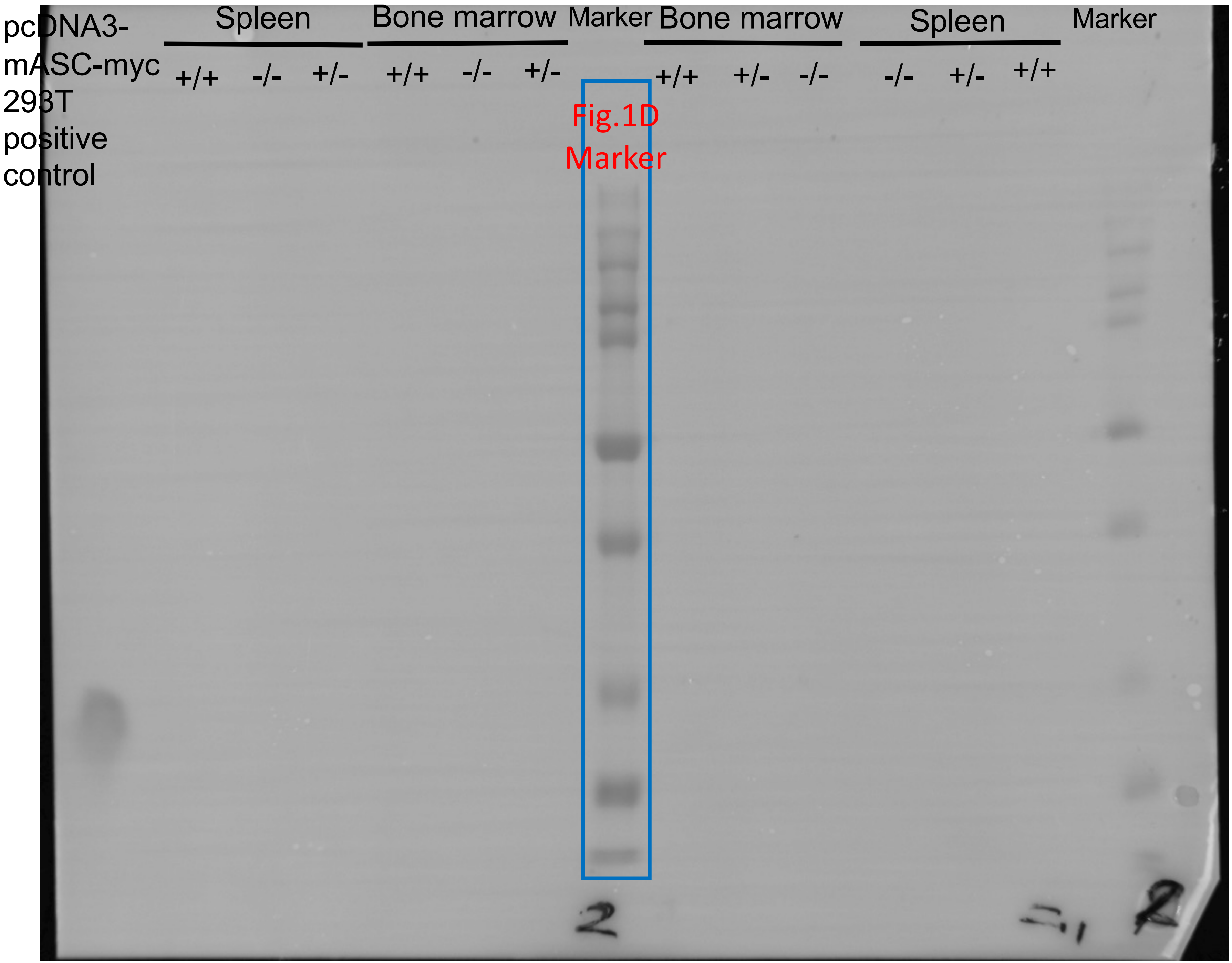

pcDNA3-mASC-myc 293T positive control
Spleen
+/+ -/- +/-
Bone marrow
+/+ -/- +/-
Marker
Fig.1D Marker
Bone marrow
+/+ +/- -/-
Spleen
-/- +/- +/+
Marker
